# Auditory time thresholds in the range of milliseconds but not seconds are impaired in ADHD

**DOI:** 10.1038/s41598-022-05425-2

**Published:** 2022-01-25

**Authors:** Giovanni Anobile, Mariaelisa Bartoli, Chiara Pfanner, Gabriele Masi, Giovanni Cioni, Francesca Tinelli

**Affiliations:** 1 Department of Neuroscience, Psychology, Pharmacology and Child Health, University of Florence, Florence, Italy; 2 Department of Developmental Neuroscience, IRCCS Fondazione Stella Maris, Pisa, Italy; 3 Department of Clinical and Experimental Medicine, University of Pisa, Pisa, Italy

**Keywords:** Paediatric research, Human behaviour, Outcomes research

## Abstract

The literature on time perception in individuals with ADHD is extensive but inconsistent, probably reflecting the use of different tasks and performances indexes. A sample of 40 children/adolescents (20 with ADHD, 20 neurotypical) was engaged in two identical psychophysical tasks measuring auditory time thresholds in the milliseconds (0.25–1 s) and seconds (0.75–3 s) ranges. Results showed a severe impairment in ADHD for milliseconds thresholds (Log10BF = 1.9). The deficit remained strong even when non-verbal IQ was regressed out and correlation with age suggests a developmental delay. In the seconds range, thresholds were indistinguishable between the two groups (Log10BF = − 0.5) and not correlated with milliseconds thresholds. Our results largely confirm previous evidence suggesting partially separate mechanisms for time perception in the ranges of milliseconds and seconds. Moreover, since the evidence suggests that time perception of milliseconds stimuli might load relatively less on cognitive control and working memory, compared to longer durations, the current results are consistent with a pure timing deficit in individuals with ADHD.

## Introduction

According to current diagnostic systems (DSM-5), attention deficit hyperactivity disorder (ADHD) is defined by pervasive and severe symptoms of inattention, hyperactivity, and impulsivity that have a direct negative impact on social, academic, or occupational functioning. ADHD children often show deficits in planning, organization and in executive functions, such as response inhibition, interference control, reasoning, hindsight, anticipation and working memory set-shifting^1^.

Along with these deficits, findings suggest perceptual disfunctions related to time processing. Time perception in ADHD has been widely investigated but the results are mixed, probably reflecting the many different methods and performance parameters. Measures of time processing includes accuracy (how far from the target) and/or precision (response variability) measured by motor reproduction, verbal estimation, discrimination, and odd-ball tasks. Moreover, time processing has been investigated across different timing ranges (from a few milliseconds to several seconds) and across sensory modalities^2^. Even if the literature and the clinical practice indicate an impaired time processing in ADHD, the highly heterogeneous results make it difficult to draw firm conclusions.

A meta-analysis considering 27 studies on ADHD children and adolescents found a significant timing deficit in ADHD with impairments in both accuracy and precision and across visual and auditory stimuli^3^. Another recent meta-analysis considering 12 studies, despite generally confirming a time deficit in ADHD, suggested that there was a stronger effect size for tasks involving relatively long intervals (5 s) and for specific tasks such as estimation and reproduction^4^. An example of how the task and the timing range could affect the results comes from the study conducted by Smith et al.^5^. The authors measured time discrimination thresholds in a sample of ADHD children by asking participants to indicate which one of two audio-visual stimuli, ranging around 1 s, lasted longer. Results revealed higher thresholds (lower precision) in children with ADHD. In contrast, no group differences were found when time processing was measured with a reproduction task or with a verbal estimation task with longer (5 s–10 s) stimuli. Toplak and Tannock^6^ also measured duration discrimination thresholds in a sample of ADHD adolescents for visual and auditory stimuli and for both short (around 200 ms) and longer (around 1 s) intervals. Results confirmed higher thresholds compared to controls, but across all the tasks. Similar impairments across vision and audition were later obtained by Rubia et al.^7^ with younger ADHD children and by Dölek et al.^8^ with adults. Plummer and Humphrey^9^ measured ADHD children’s timing performance with a motor reproduction task. Children were asked to reproduce, by key press, the duration of visual, auditory, or audio-visual stimuli ranging from 1 to 60 s. Results showed higher errors in ADHD across all the conditions. Radonovich and Mostofsky^10^ measured ADHD duration discrimination thresholds for short (around 0.5 s) and longer (around 4 s) auditory stimuli. At odds with some of the previous studies, the results showed no deficits for the shorter stimuli but poorer performance for longer intervals. Gooch et al.^11^ measured auditory time discrimination thresholds in ADHD by asking children to detect which of three sounds was different in duration (odd-one-out task) with stimuli ranging from 400 ms to 1 s. The results revealed higher thresholds (lower precision) in children with ADHD, compared to controls. The same results were obtained with a motor reproduction task for longer visual stimuli (from 2 to 10 s). Barkley^12^ measured time perception with a verbal estimation and a reproduction task testing the same visual stimuli, lasting from 2 to 60 s. The results revealed poorer time perception (accuracy) for the reproduction, but not for the estimation, task. Two independent studies employing similar visual time intervals found the same pattern of results^13,14^.

Interestingly, psychophysical and pharmacological studies indicate that the perception of relatively long stimuli requires and loads on cognitive control as well as working memory, while relatively shorter intervals (< 1 s) might be automatically processed and targeting a “pure” sense of time^15–17^. A more recent study using imaging techniques showed that although there are many brain areas encoding both short (milliseconds) and longer (seconds) durations, short durations elicit relatively more activation in the parietal cortex^18^. The authors suggest that this higher activation reflects a higher involvement of attentional resources when encoding short stimuli. However, the distinction between the use or non-use of attentional control as a function of stimulus duration is not clear-cut and there is also evidence for the involvement of cognitive resources in intervals in the millisecond range, leaving this issue largely open^19^.

Overall, these and many other investigations suggest a time processing deficit in people with ADHD, with potentially relevant clinical and practical implications^20^. However, given the heterogeneity of results, further investigation is needed. To this aim, 20 children/adolescents with ADHD were engaged in a testing protocol in which we psychophysically measured auditory time thresholds in the milliseconds (0.5 s) and seconds (1.5 s) ranges. To avoid methodological confounds, the same psychophysical technique (categorization task, see methods) was used for both timing ranges. The performance on these tasks was compared to those obtained from 20 age-matched neurotypical controls. As many studies have found time perception deficits in children and adolescents with ADHD, we expected higher thresholds, on at least one of the timing ranges tested here. As perception of short stimuli has been suggested to depend relatively less on cognitive control and working memory, compared to longer durations, a specific deficit for short stimuli would suggest a pure time deficit.

## Materials and methods

### Participants

Forty children/adolescents participated in this study: 20 with ADHD (6 female, 14 males, mean age = 11.2 year old, age range 8–16) and 20 neurotypical (11 female, 9 males, mean = 11.2 year old, range 8.1–16.2). Individuals with ADHD were enrolled from the Stella Maris Foundation Institute in Pisa, a main center for ADHD care in Italy. ADHD inclusion criteria were: clinical diagnosis of ADHD based on DSM-5, a total intelligence quotient (TIQ), evaluated with the Wechsler Intelligence Scale for Children-IV^21^ above 75, no neurological or sensory deficits, no psychiatric comorbidities, no current or past pharmacological treatment. Three children with ADHD met the criteria for a diagnosis of developmental dyslexia. Non-verbal reasoning skills were computed by a combined index of WISC-IV measuring Visual Perceptual Reasoning (IRP). The IQ of four ADHD participants was measured by an external independent institute and, for those participants, we were unable to calculate IRP. ADHD symptoms were measured by Conners Rating Scale (parent version). General clinical symptoms were measured by the Clinical Global Impression—Severity scale (CGI) and the Children Global Assessment Scale (CGAS). Detailed information about the group with ADHD is reported in Table 1.

**Table1:** Descriptive characteristics of the ADHD group.

Participants	Sex	Age	TIQ	IRP z-score	Subtype	CGI-S	CGAS	C1	C2	C3	C4
1	M	15 years 6 months	103	1.13	1	3	60–51	n.a	n.a	n.a	n.a
2	F	9 years 6 months	90	− 0.73	1	2	70–61	45	60	52	65
3	F	12 years 3 months	88	− 1.2	3	2	70–61	42	68	51	67
4	M	9 years 3 months	94	− 0.4	3	4	50–41	77	73	63	80
5	M	14 years 11 months	97	− 1.2	3 + dd	4	50–41	77	75	61	75
6	M	13 years 3 months	125	n.a	3	2	70–61	55	49	35	56
7	M	10 years 5 months	114	n.a	1	2	60–51	74	70	70	72
8	M	8 years 4 months	91	− 0.13	3	4	60–51	78	75	70	80
9	M	11 years	110	n.a	3	5	50–41	71	75	80	80
10	M	9 years 1 months	114	1.26	3	4	60–51	80	75	63	80
11	M	12 years 6 months	88	n.a	3	2	70–61	60	53	57	65
12	F	11 years 1 months	98	− 1.2	3	4	50–41	n.a	n.a	n.a	n.a
13	M	11 years 1 months	100	0	3	3	60–51	55	50	47	61
14	F	9 years 8 months	105	− 0.13	3	3	60–51	45	56	52	63
15	F	12 years 1 months	92	0.13	3 + dd	4	60–51	n.a	n.a	n.a	n.a
16	M	11 years 9 months	77	− 1	1	3	70–61	70	100	56	100
17	M	12 years 6 months	101	− 1.2	3	6	60–51	n.a	n.a	n.a	n.a
18	M	11 years 6 months	102	0.4	1	6	60–51	64	63	61	66
19	M	8 years 7 months	107	0.73	3	5	50–41	64	66	70	n.a
20	F	10 years	96	1.13	3 + dd	6	60–51	49	39	48	13

*TIQ* Total Intelligence Quotient from WISC-IV, *IRP z-score* Visual Perceptual Reasoning from WISC-IV; *Subtype* 1 inattentive, 3 combined; *dd* developmental dyslexia, *CGI* Clinical Global Impression – Severity scale, *CGAS* Children Global Assessment Scale, *C1* Conners parents oppositivity, *C2* Conners parents inattanetion, *C3* Conners parents hyperactivity, *C4* Conners parents ADHD index, *n.a.* not available.

The participants with ADHD were compared to a neurotypical group of age matched children/adolescents. The inclusion criteria for the control group were: no medical history, negative neuro-psychiatric exam and no learning difficulties (reported by parents) and IQ (evaluated with by Raven Colored Progressive Matrix-CPM or Progressive Matrix-PM, depending on chronological age) > 5° percentile. The study was approved by the Ethics Committee of the Meyer’s Hospital (n. 248/2020 ID-DNATN "Attention, Time and Numeracy in children and adolescents with neurodevelopmental disorders")*.* Informed parental consent was obtained for each participant before the study. All experiments were performed in accordance with relevant guidelines and regulation.

### Time perception

**Figure 1:**
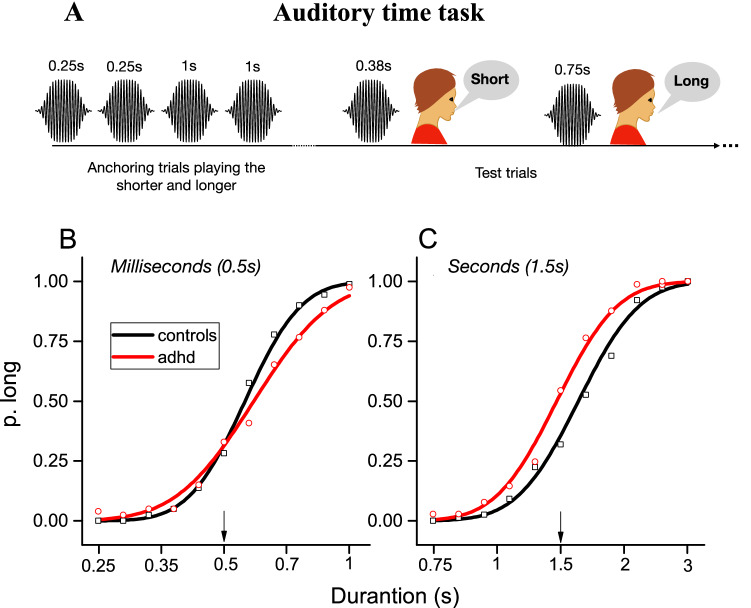
(**A**) A block consisted of four initial “anchoring” trials in which the shorter (0.25 s or 0.75 s depending on the condition) and the longer (1 s or 3 s depending on the condition) stimuli were presented (participants were told that those sounds correspond to the range extremes). After this phase, the testing phase started, and participants were asked to categorize as “long or short” a sound randomly drawn from a pre-defined distribution. (**B**,**C**) Psychometric functions (aggregate data) for controls (black, squares) and participants with ADHD (red, circles) for the milliseconds (**B**) and seconds (**C**) ranges.

Time sensory thresholds were psychophysically measured with an auditory categorization task (Fig. 1A). On each trial, children listened to a single sound (500 Hz, 80 dB pure tone) and were asked to categorize it as “long” or “short”. Before the testing phase, four initial “anchoring” trials were provided; the lower and longer time durations were played twice each and the children were told that those sounds corresponded to the range extremes (no responses were required). In separate sessions (lasting around 4 min each), we measured two different timing regimes, one centered (geometric mean) around 0.5 s containing stimuli in the milliseconds range (from 0.25 s to 1 s, milliseconds hereafter), and one centered (geometric mean) around 1.5 with most of the stimuli belonging to the seconds range (from 0.75 to 3 s, seconds hereafter). Each time range was divided into 11 equal steps spanning 1 octave above and one below the geometric mean of that specific range. The stimuli in the 1.5 s distribution were: 0.75, 0.86, 1, 1.13, 1.3, 1.5, 1.72, 1.98, 2.27, 2.61, 3 s. The stimuli in the 0.5 s distribution were: 0.25, 0.28, 0.33, 0.38, 0.43, 0.5, 0.57, 0.66, 0.75, 0.87, 1 s. In a single session, each duration was tested 4 times (randomly selected trial-by-trail) for a total of 44 trials for each range. Participants responded verbally (“long” or “short”), without any time pressure and the response was registered by the administrator with an appropriate key press. The proportion of “long” responses were plotted against the stimuli duration (in log scale) and fitted with a cumulative Gaussian error function. The 50% point of the fit provided an estimate of the point of subjective equality (PSE). The difference in duration between the 50% and 75% points gives the just notable difference (JND), which was used to estimate Weber Fractions (10^JND-1), a dimension free index of sensory precision.

### Data analysis

Data were analyzed by Repeated Measures Analyses of Variance (RM-ANOVA), Analysis of covariance (ANCOVA), t-test and Pearson correlations and α values corrected for multiple comparisons when necessary (Bonferroni correction). Frequentist statistics were supplemented with Bayesian statistics, calculating Bayes Factors, the ratio of the likelihood of the alternative to the null hypothesis, and reporting them as base ten logarithms (Log10 Bayes Factors, LBF). For RM-ANOVA and ANCOVA with report LBF_inclusion_ indicating how much the data are likely to occur from a model including that specific factor (or interaction), compared to models not including them. By convention, LBF > 0.5 is considered substantial evidence in favour of the alternative hypothesis (difference between groups in this case) and LBF < − 0.5 substantial evidence for the null hypothesis (no difference). Absolute values greater than 1 are considered strong evidence, and those greater than 2 as definitive evidence.

To make the ADHD Visual Perceptual Reasoning (IRP) index comparable to the non-verbal reasoning skills measured by Raven matrices on the control group, the indexes were both converted into z-scores (according to the normative age-standardized data provided by the tests manuals). For technical reasons we did not collect data for two participants (one control and one ADHD) in the seconds timing task. Missing data were left empty and excluded analysis by analysis. Effects sizes were reported as Cohen-d and η^2^. Data were analyzed by JASP (Version 0.8.6), SPSS (Version 25) and R (Version 4.0.2) software.

## Results

### Demographical data

The ADHD and control groups did not differ in age (t_(38)_ = 0.035, p = 0.97, d = 0.011, LBF = − 0.52) and male/female ratio (X^2^ = 0.93, p = 0.33). Non-verbal reasoning skills were slightly lower in the group with ADHD (t_(32)_ = 1.8, p = 0.083, d = 0.6, LBF = 0).

### Groups difference on time perception sensory precision

All participants were well able to perform the psychophysical timing task (depicted in Fig. 1A) producing ordered psychometric functions. Figure 1B,C shows psychometric functions obtained aggregating all the data together across participants. It is evident, even by inspection, that for the task measuring time perception in the milliseconds range (B), the psychometric function of the control participants (black) was steeper than that produced by the sample with ADHD (red). This difference indicates less precision (higher thresholds, Weber Fractions) in individuals with ADHD. Regarding the seconds range (C) the psychometric functions have similar slopes between groups, indicating similar sensory precision levels.

**Figure 2:**
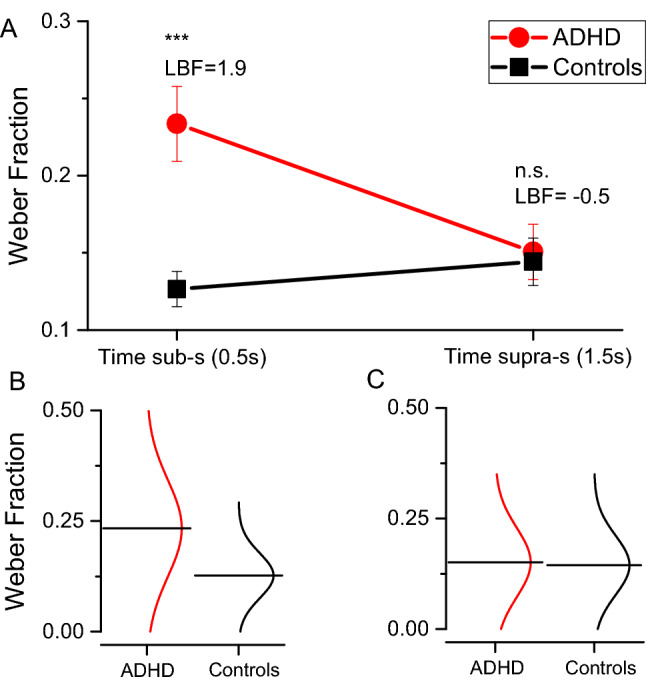
(**A**) Between participants average time thresholds divided by the two groups (ADHD: red circles and Controls: black squares). Error bars are standard errors of the mean; ***p < 0.001; n.s. not statistically different. (**B**–**D**) Individual data reporting time thresholds distributions. Horizontal lines report data average.

The fitting procedure was applied to the data provided by each participant (see Table 2 for descriptive statistics). Figure 2 reports between participants average thresholds (Weber Fractions, Wf) separately for the two perceptual tasks, while single subject data are reported in Fig. 2B,C. From visual inspection, it is evident that only the time perceptual thresholds measured for short (0.5 s) auditory stimuli were impaired in participants with ADHD, with a clear interaction between tasks and groups.

**Table 2:** Descriptive Statistics on time thresholds (Weber Fraction).

Tasks	Groups	N	Mean (SD)	p-value	Cohen's d	LBF
Time milliseconds range (0.5 s)	ADHD	20	0.23 (0.1)	< 0.001***	1.26	1.9
	Controls	20	0.12 (0.05)			
Time seconds range (1.5 s)	ADHD	19	0.15 (0.07)	0.79	0.08	–0.5
	Controls	19	0.14 (0.06)			

Two tailed t-tests, α Bonferroni corrected 0.05/2 = 0.025.

A RM ANOVA with task (2 levels: Wf 0.5, Wf 1.5) as repeated measures factor and group (2 levels: ADHD, controls) as between participants factor, revealed a significant effect of task (F_(1,36)_ = 7.38, p = 0.01, η^2^ = 0.12, LBF_incl_ = 2.31), suggesting different thresholds across tasks. Crucially the task*group interaction was highly statistically significant (F_(1,36)_ = 18.04, p < 0.001, η^2^ = 0.29, LBF_incl_ = 2.59) indicating that the groups performed differently across the tasks. Post-hoc analyses confirmed that time thresholds for milliseconds (0.5 s) stimuli were higher in the ADHD group compared to controls (t_(38)_ = 3.98, p < 0.001, d = 1.26, LBF = 1.9). On this task, ADHD thresholds were, on average, almost double compared to controls (Wf = 0.23 and 0.12 for ADHD and controls respectively), indicating a severe impairment. Time thresholds for seconds (1.5 s) stimuli (t_(36)_ = 0.26, p = 0.79, d = 0.08, LBF = –0.5) were statistically indistinguishable between the groups.

To explore the specificity of the impairment found for the time perception task in the milliseconds range, we ran a separate ANCOVA with time thresholds as the dependent variable, groups (ADHD, controls) as the fixed factor and age, non-verbal reasoning, and sex as covariates. Even when partialling out the effect of these covariates, the result remained unchanged with a significant effect of the group (F_(1,29)_ = 20.11, p < 0.001, η^2^ = 0.33, LBF_incl_ = 2.78).

To check the discriminant power of the milliseconds auditory time thresholds, we ran a linear discriminant analysis with the group as the dependent variable and time thresholds (0.5 s Wf) as independent variable. The results revealed 72.5% of cases correctly classified. The sensitivity was 60% while specificity was 85%. As a sanity check, the same analysis on seconds stimuli thresholds (Wf) provides a near to chance level (53%) classification.

### Developmental trajectories

**Figure 3:**
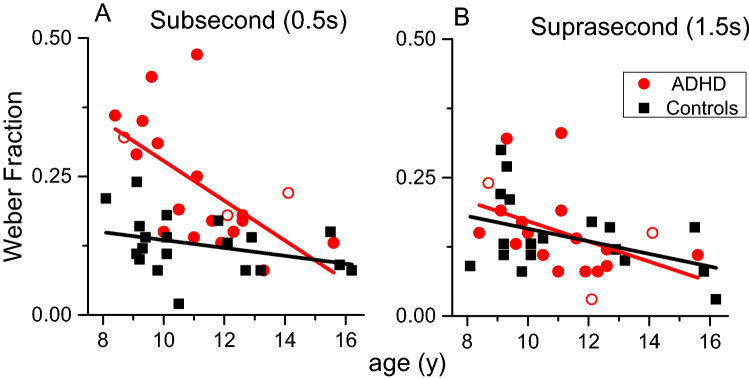
Auditory time thresholds for stimuli in the milliseconds (**A**) and seconds (**B**) ranges as a function of age divided by the two groups (ADHD: red filled circles, ADHD + dyslexia: red open circles, Controls: black squares).

To investigate whether the deficit was stable across the age range, we studied the developmental trajectories. Time thresholds for seconds stimuli had a similar and not significant dependency with age across both groups (ADHD: r = − 0.43, p = 0.062, LBF = 0.15, controls: r = − 0.42, p = 0.068, LBF = 0.12), suggesting that both were near to a developmental plateau. The developmental trajectories of time thresholds in the milliseconds range were, in contrast, different between the groups. While the controls had reached an almost full developmental stage (r = − 0.34, p = 0.14, LBF = − 0.11), the age dependence for participants with ADHD was steeper (r = − 0.62, p = 0.003, LBF = 1.2), suggesting a different developmental trend (Fig. 3). Confirming partially independent mechanisms, regressing out age, thresholds for milliseconds and seconds stimuli were not correlated with each other (ADHD: r_partial_ = 0.367, p = 0.134, LBF = 0.09 ; controls: r_partial_ = 0.287, p = 0.25, LBF = –0.07).

### Correlations with clinical symptoms

Within the sample with ADHD, we ran correlations and between time thresholds (milliseconds and seconds) and both general (CGI, CGAS, see Table 1) and specific clinical symptoms (the four parents Conners indexes, see Table 1 for details). For the CGAS test, which provides range scores, we transformed the ranges into categorical values reflecting the symptoms severity (following the test manual: from 1 to 10 with one indicating no symptoms and 10 indicating very severe symptoms). The analyses revealed no meaningful correlations (all p > 0.05, min LBF = − 0.55, max LBF = 0.3).

### Perceptual task reliability

It is theoretically possible that the different pattern of results provided by the two time tasks results from different reliability levels. To test this possibility, we measured and compared the reliability of the two psychophysical tasks. Following previous studies^22^, we used a “sample-with-replacement” bootstrap technique^23^. For each participant, we calculated two separate thresholds in each task (0.5 s or 1.5 s), using a random sample of the data (44 trials, sampled with replacement), and then computed the correlation between those two measures, across participants. The process was reiterated 1,000 times. We found that mean correlations for milliseconds (0.5 s) and seconds (1.5 s) stimuli were very similar (Pearson’s r = 0.68, r = 0.64, respectively) and not statistically different (bootstrap sign-test p = 0.4). This last control rules out the possibility that the different pattern of results was generated by different reliability levels.

### Contextual effects

**Figure 4:**
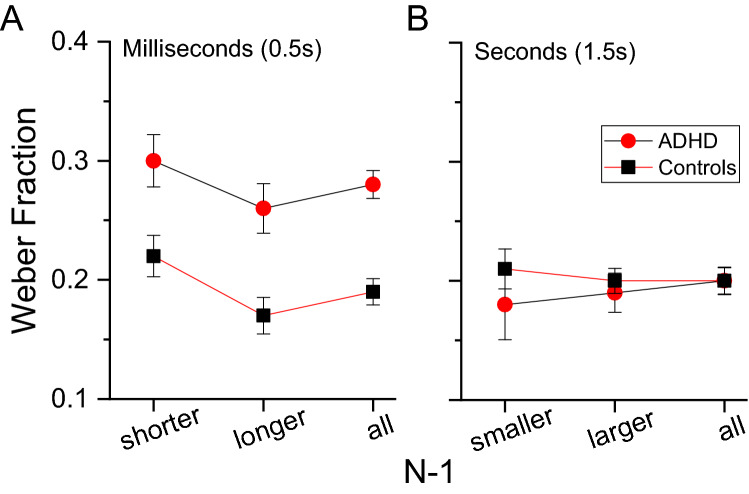
Auditory time thresholds for stimuli in the milliseconds (**A**) and seconds (**B**) ranges measured on all trials (all) or on data sorted as a function of the preceding stimulus duration (N-1) that could be shorter or longer compared to the stimulus judged in the current trial.

The paradigm used to measure time thresholds requires the ability to perceive both the stimuli mean and range extremes of the set. The group with ADHD could have had excessive contextual effects, which would have inflated thresholds. To check for this possibility, we measured (on aggregate data) the PSEs and thresholds as a function of the magnitude of the preceding stimulus (N-1). To this aim, separately for the two groups and the two tasks, we sorted the aggregated data into two categories in which the preceding stimulus (N-1) was shorter or longer than the stimulus tested in the current trial. The data were then fitted by psychometric functions providing PSEs and thresholds (Weber fraction). The analyses releveled very small effects on PSEs for both groups (ADHD 1.5 s = shorter 1.4 s, longer 1.5 s; ADHD 0.5 s = shorter 0.56 s, longer 0.61 s; controls 1.5 s = shorter 1.6 s, longer 1.6 s; controls 0.5 s = shorter 0.55 s, longer 0.56 s), suggesting similar contextual effects. More importantly, for both subdivisions (shorter, longer), the difference between groups for thresholds in the milliseconds condition remained evident and constant (Fig. 4) confirming similar contextual effects.

### Time perception accuracy

**Figure 5:**
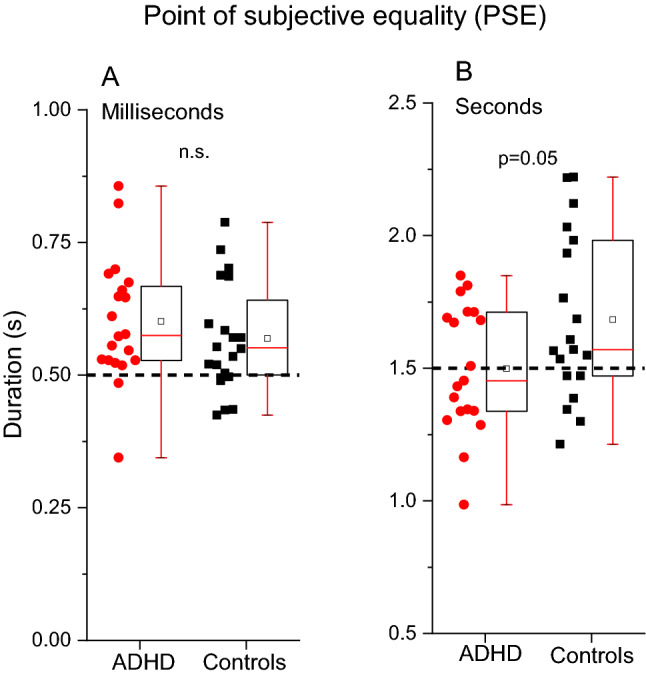
Box plots reporting point of subjective equality (PSEs) for the auditory time task in the millisecond (**A**) and seconds (**B**) ranges for the two groups of participants. Dotted lines report the physical value of the reference stimuli (0.5 s and 1.5 s). Symbols within the boxes reports median (line) and mean (open square) values.

After exploring the differences between groups in terms of sensory precision, we then analyzed estimations accuracy (PSE: Point of Subjective Equality, see Fig. 5 and Table 3). A RM ANOVA with task (PSEs time 0.5 s, PSEs time 1.5 s) as repeated measures factor and group (ADHD, controls) as between participants factor revealed an obvious significant effect of task (F_(2,36)_ = 420.7, p < 0.01, η^2^ = 0.91) suggesting that PSEs change as a function of the task. Importantly, the task*group interaction was statistically significant (F_(2,36)_ = 5.03, p = 0.031, η^2^ = 0.01) indicating that the groups performed differently across the tasks. The between factor “group” was not statistically significant (F_(1,36)_ = 2.3, p = 0.13, η^2^ = 0.06) suggesting that, on average, the two groups performed similarly. To explore the interaction, we ran a series of post-hoc t-tests (α Bonferroni corrected = 0.025). In the range of milliseconds, the analysis revealed no differences between groups (t(38) = 0.91, p = 0.37, Cohen’s d = 0.28). In the range of seconds the group with ADHD, compared to the controls, showed a tendency to overestimate durations (t(36) = 2.03, p = 0.05, Cohen’s d = 0.65).

**Table 3:** Descriptive statistics: point of subjective equality (PSEs).

Tasks	Groups	N	Mean (SD)	p-value	Cohen's d
Time milliseconds range (reference: 0.5 s)	ADHD	19	0.6 (0.12)	0.37	0.28
	Controls	19	0.57 (0.1)		
Time seconds range (reference: 1.5 s)	ADHD	20	1.49 (0.24)	0.05	0.66
	Controls	20	1.68 (0.31)		

Two tailed t-tests, α Bonferroni corrected 0.05/2 = 0.025.

## Discussion

We found that children/adolescents with ADHD, compared to neurotypicals, had a severe sensory precision deficit in perceiving auditory stimuli in the milliseconds range (0.25-1 s). Time thresholds for relatively longer stimuli (0.75-3 s) were unimpaired. Thresholds did not correlate between each other, and only thresholds for milliseconds durations showed a different developmental trajectory between groups, suggesting a developmental delay in participants with ADHD. Two control analyses ruled out the possibility that the pattern of results was driven by different tasks reliability levels and different use of contextual effects between groups. Consistent with previous studies^24^, time processing abnormalities were equally reported in the various presentations of ADHD.

Time is not a unitary concept but encompasses many measurement scales, from a few milliseconds to several days. Even if there is not a defined boundary classifying a duration as “short” or “long”, one of the most classic distinctions is that between milliseconds and seconds stimuli. Clear dissociations have been previously found by pharmacological studies. Rammsayer et al.^25^ found that administering ethanol impaired auditory temporal discrimination thresholds for long (1 s) but not shorter (50 ms) intervals. A similar pharmacological dissociation was later found by Rammsayer^17^ showing that auditory temporal processing of long durations (1 s) was significantly impaired by administration of haloperiodol (a dopamine receptor antagonist) and midazolam (benzodiazepine), whereas processing of extremely brief intervals (50 ms) was only affected by haloperidol. The authors suggested that temporal processing of longer intervals is mediated by working-memory functions, while temporal processing of intervals in the range of milliseconds is more dependent on the effective level of dopaminergic activity in the basal ganglia. Psychophysical experiments also supported this differentiation. For example, it has been demonstrated that increasing cognitive load by dual-task procedures deteriorates discrimination thresholds for relatively long (1 s), but not short (50 ms) auditory stimuli, suggesting that the encoding of longer intervals requires cognitive control and working memory, while relatively shorter intervals are automatically processed^15,16^. However, is worth mentioning that the link between time perception and cognition remains poorly understood and there is also evidence challenging whether or not cognitive control and working memory play a role merely based on stimulus length. For example, Holm et al.^19^ found that task cognitive and working memory loads both worsened repetitive motor timing (tapping) for durations in the millisecond range, more than in the seconds range. By a factor analyses approach Rammsayer et al. demonstrated that, while the model assuming two mechanisms underlying the processing of intervals in the seconds and the milliseconds ranges might be more appropriate, these two mechanisms are functionally overlapped, sharing 77% common variance^26^. Moreover, imaging studies suggest that, in addition to the duration of the stimuli, other factors such as the use of movement to define a temporal estimate and the continuity and predictability of the task may influence the engagement of different timing mechanisms, making the picture more complex than previously thought^27^.

By showing a specific impairment of auditory time thresholds for relatively short (0.5 s) but not longer (1.5 s) intervals in ADHD, the current results fit well with the idea of different mechanisms for those regimes. The null correlation between thresholds across the two-timing regimes is also consistent with this hypothesis. Given that ADHD is often associated with deficits in cognitive control and working memory, it seems counterintuitive that timing thresholds for automatic, but not those under cognitive control, were impaired in our sample with ADHD, suggesting a pure time perception deficit. Leaving aside whether or not cognitive resources are engaged depending on the duration of the stimuli, an issue that has not been directly tested here, the current results (together with much previous evidence) are difficult to explain, suggesting a unique system for timing perception.

It is worth noting that our results are opposite to those found by Radonovich et al.^10^. In that study the authors measured auditory discrimination thresholds in controls and in children/adolescents with ADHD with short (550 ms) and longer (4 s) intervals and their results demonstrated worse thresholds for long but not short intervals. Although we do not have a definitive explanation to account for this difference, the different tasks could, even partially, account for that. The paradigm used by Radonovich et al. presented two pairs of tones, one with fixed delay and the other with variable delays, and participants were asked to report whether the second delay was shorter or longer than the first. Our paradigm required the presentation and categorization of a single tone. We find it reasonable to speculate that the Radonovich et al. task, compared to the task used here, would charge relatively more additional resources such as working memory and/or attention, which may account for the different pattern of results.

Regarding brain areas involved in the perception of milliseconds and seconds intervals, the literature, despite suggesting different networks, is not definitive. Mangles et al.^28^ compared auditory time discrimination thresholds between controls and patients with focal lesions in the frontal cortex or in the cerebellum. The results indicate that frontal lesions impaired timing thresholds for long (4 s) but not short (0.4 s) intervals, while cerebellar lesions impaired both. Harrington et al.^29^, however, cast doubts on the involvement of the cerebellum in the auditory time perception of milliseconds intervals (0.3 s, 0.6 s), with patients with cerebellar lesions showing similar thresholds compared to controls. On the other hand, cerebellar lesions have been demonstrated to have detrimental effects on visual time thresholds for longer (8–21 s) stimuli^30^. As mentioned before, basal ganglia have also been found to play a role in time perception. Gouvêa et al.^31^ trained rats to categorize sounds as belonging to a long or short category. Animals made few errors when categorizing the shortest and longest intervals (the extremes), but performance become worse for intervals near to the 1.5 s categorical boundary (range: 0.6–2.4 s). Recording from populations of single striatal neurons, the authors found cells firing at different times within the interval period, suggesting the existence of short and long preferring neurons. The causal role of striatal neurons was also demonstrated by injecting muscimol. As a result, the duration thresholds worsened significantly, compared to the control group injected with saline. The parietal cortex has also been shown to play a critical role in time perception^32–35^. Hayashi et al.^36^ correlated time discrimination thresholds measured in a sample of neurotypical adults, with gray matter (GM) volume in different parts of cortical and subcortical areas. Results showed that GM volume in the cerebellum but not in the parietal cortex correlated with milliseconds stimuli thresholds whilst, contrarily, GM volume in the parietal cortex but not in the cerebellum correlated with seconds stimuli thresholds. Moreover (as in the current study) threshold for milliseconds and seconds stimuli did not correlate with each other. In the same line, a meta-analysis of imaging studies also suggests that the parietal cortex, compared to the cerebellum, is more likely activated by seconds stimuli^27^. With the current psychophysical data, we cannot say much about the brain networks involved in milliseconds and seconds timing, but our findings are largely in line with the idea that those two functions engage distinct neural mechanisms.

Another interesting point emerges from the current results. The analysis of developmental trajectories (Fig. 3A) suggests that, rather than a generalized deficit across all ages, there could be a developmental delay for milliseconds thresholds in ADHD. Despite having few participants in the higher age range, the results seem to suggest that above 12/13 years old, the difference between groups would gradually decrease. Future studies might replicate this finding and expand the age range to quantitatively define the magnitude of such a possible developmental delay.

Together with a sensory precision deficit, the existing literature has also shown that people with ADHD are more likely, compared to neurotypicals, to commit overestimation errors, suggesting a faster “internal clock”^37,38^. Our results are in line with this idea. In the task testing durations in the seconds range, participants with ADHD provided more "long" judgements compared to controls, leading to a leftward shift of the psychometric curve. This bias indicates a duration overestimation, probably arising from a faster internal clock. In the task testing durations in the milliseconds range, there was no difference between groups, reinforcing the idea of partially independent mechanisms for short (ms) and longer (secs) intervals and replicating previous evidence^37^.

## Conclusions

A precise timing perception in the millisecond scale is crucial in perceiving and acting on the continuously changing environment. It has been suggested that this ability is automatic and allows complex human behaviours including speech perception and speech performance, music, driving, and many sports. The results of this study showed that children and adolescents with ADHD have a severe precision (thresholds) deficit in the duration perception of milliseconds (around 0.5 s) auditory stimuli. Time perception for relatively longer and more cognitively controlled stimuli was unimpaired. Although, with the current results, we cannot explain why the temporal perception of short stimuli is impaired in our sample with ADHD, it is surprising how such a simple and fast (4-min) psychophysical test succeeds in differentiating the two groups (LBF = 1.9). Moreover, while timing thresholds had a relatively low sensitivity power (60%), they turned out to have a good specificity (85%) level and were able to correctly classify 72.5% of cases. Finally, the timing thresholds deficit was resistant to the statistical controls of general but important covariates such as age, sex and non-verbal IQ, promoting it as a potential easy-to-administer tool for future studies. A better comprehension of time perception abnormalities in ADHD may give new insights in the neurological bases of the disorder. Furthermore, it may have relevant diagnostic and treatment implications. Understanding the functional consequences of these specific deficits in everyday life, in combination with other ADHD symptoms (i.e. impulsivity), may help to focus interventions on more specific goals, improving the clinical care of youth with ADHD. Moreover, as ADHD and autism might represent a continuum, sharing many pathophysiological features^39^, it would be interesting and potentially relevant to test whether both clinical conditions also share a similar pattern of deficits in time perception.
